# Draft Genome Sequence of Streptomyces xinghaiensis (*fradiae*) OlgR, a Strain Resistant to Oligomycin A

**DOI:** 10.1128/MRA.01531-18

**Published:** 2019-01-10

**Authors:** Olga B. Bekker, Aleksey A. Vatlin, Natalia V. Zakharevich, Ludmila N. Lysenkova, Andrey E. Shchekotikhin, Valery N. Danilenko

**Affiliations:** a Vavilov Institute of General Genetics, Russian Academy of Sciences, Moscow, Russia; b Gauze Institute of New Antibiotics, Moscow, Russia; Queens College

## Abstract

We report a draft genome sequence of Streptomyces xinghaiensis (*fradiae*) OlgR, which is resistant to oligomycin A. This mutant strain is derived from S. xinghaiensis OlgR2.100, which is resistant to (33*S*)-azido-33-deoxyoligomycin A. We have identified single nucleotide polymorphisms (SNPs) in 7 genes, which may lead to oligomycin A resistance.

## Abstract (precis)

We report a draft genome sequence of Streptomyces xinghaiensis (*fradiae*) OlgR, which is resistant to oligomycin A. This mutant strain is derived from S. xinghaiensis OlgR2.100, which is resistant to (33*S*)-azido-33-deoxyoligomycin A.

## ANNOUNCEMENT

Streptomyces xinghaiensis strain ATCC 19609 (isolated from soil), which produces tylosin (1), is hypersensitive to most known antibiotics, including oligomycin A (MIC, 0.001 µM) (2) and its derivatives. Attempts to obtain a mutant strain of S. xinghaiensis ATCC 19609 resistant to oligomycin A were unsuccessful because oligomycin A has several biotargets in the cell (3). Previously, we obtained mutant strain S. xinghaiensis OlgR2, which is resistant to (33*S*)-azido-33-deoxyoligomycin A, an oligomycin A derivative modified by a macrolactone ring in the C-33 position (3), which has a central function in the binding of oligomycin A and the biotarget. The next step was to obtain the mutant strain S. xinghaiensis OlgR, resistant to oligomycin A, to search for additional oligomycin A biotargets. The frequency of obtaining mutants was 2 × 10^9^. The mutant was selected on solid medium containing 10 µM oligomycin A (MIC, 0.001 µM).

S. xinghaiensis spores were collected from an agar plate inoculated in liquid yeast extract-malt extract (YEME) medium with oligomycin A (10 µM) for 48 h at 28°C and 250 rpm. Genomic DNA was extracted from S. xinghaiensis using the standard techniques for *Streptomyces* (4). The isolated DNA was purified using the GenElute bacterial genomic DNA kit (Sigma). The NEBNext Ultra II protocol was used to prepare a paired-end library after DNA fragmentation on a Covaris S220 instrument. The raw sequencing data were obtained using an Illumina MiSeq version 2 instrument (500 cycles). The library had reads of 240 bp (forward) and 225 bp (reverse) and a mean coverage of 253×. The quality of reads was controlled by FastQC and Trimmomatic programs. Mapping of reads on the reference genome was performed using Bowtie 2. The number of reads was 1,666,063 × 2 (Sequence Read Archive accession no. SRR8242143). All reads were assembled into an initial draft genome of 7,734,742 nucleotides by SPAdes version 3.11. The default software settings were used. The resulting draft genome sequence consists of 150 contigs (largest contig, 1,037,856 bp; contig *N*_50_, 459,751 bp; overall GC content, 72.70%). The automatic functional annotation results were obtained using the NCBI Prokaryotic Genome Annotation Pipeline (https://www.ncbi.nlm.nih.gov/genomes/static/Pipeline.html) version 4.5. The S. xinghaiensis (*fradiae*) OlgR genome contains 6,666 predicted genes and 89 RNA genes (21 rRNA genes [4 5S, 6 16S, and 11 23S], 3 noncoding RNAs [ncRNAs], and 65 tRNAs). A total of 6,577 coding sequences (CDS), 3 clustered regularly interspaced short palindromic repeats (CRISPRs), and 78 frameshifted genes were predicted using the NCBI Prokaryotic Genome Automatic Annotation Pipeline (PGAAP). In addition, 7 insertion sequence (IS) elements were found.

During bioinformatics analysis of the S. xinghaiensis OlgR genome, we found seven single nucleotide polymorphisms (SNPs), ATP synthase F0 subunit A (WP_109321387) (oligomycin A biotarget [5]), helicase (WP_070159460) (involved in the mechanism of formation of resistance to oligomycin A and its derivatives [6]), histidine kinase (WP_019709751), DNA ligase (WP_109321419), RecB (WP_109321400), and two hypothetical proteins (WP_109321402 and WP_070159612). The first four SNPs were also found in the previous strain by Sanger sequencing (Streptomyces fradiae OlgR2).

Thus, we detected 3 additional mutations that may be involved in the formation of oligomycin A resistance in the mutant strain S. xinghaiensis OlgR. Participation in the formation of oligomycin resistance is the subject of our further research.

### Data availability.

Raw reads for these projects were assigned accession no. SRP170678 in the NCBI Sequence Read Archive. This whole-genome shotgun project has been deposited at DDBJ/EMBL/GenBank under the accession no. QFBD00000000. The version described in this paper is the second version, QFBD02000000.
